# Socially induced false memories in the absence of misinformation

**DOI:** 10.1038/s41598-022-11749-w

**Published:** 2022-05-11

**Authors:** Ullrich Wagner, Pascal Schlechter, Gerald Echterhoff

**Affiliations:** 1 Department of Psychology, University of Münster, Fliednerstr. 21, 48149 Münster, Germany; 2 Department of Psychiatry, University of Cambridge, Cambridge, UK

**Keywords:** Psychology, Human behaviour

## Abstract

Ample evidence shows that post-encoding misinformation from others can induce false memories. Here, we demonstrate in two experiments a new, tacit form of socially generated false memories, resulting from interpersonal co-monitoring at encoding without communication of misinformation. Pairs of participants jointly viewed semantically coherent word lists, presented successively in blue, green, or red letters. Each individual was instructed to memorize words presented in one of the colors. One color remained unassigned (control condition). Participants (total *N* = 113) reported more false memories for non-presented words (lures) semantically related to partner-assigned than to control lists, although both list types were equally irrelevant to their own task. Notably, this effect also persisted for particularly rich memories. These findings show for the first time that social induction of false memories, even subjectively rich ones, does not necessarily require communication of deceptive information. This has important implications both theoretically and practically (e.g., in forensic contexts).

## Introduction

Human memory is highly susceptible to social influence. Given that humans are members of an “ultrasocial” species^1^, our memory contents are constantly shaped by social interaction and communication^2,3^. However, such influences not always strengthen veridical memories, but can also be sources of false memories, i.e., memories for events that did not actually happen^4,5^, exemplified by the famous misinformation effect^6^, memory conformity^7^ and social contagion of memory^8,9^. A chief tool for exerting social influence are suggestive techniques, which can create even rich and vivid false memories^10,11^. Such influence can have serious, unwanted consequences, such as biased assessments of past events, suboptimal decision-making, and unfair judgments of actors, even the imprisonment of innocent people^12–15^.

Notably, these previous demonstrations of socially induced false memories rely on communication of misinformation presented after the initial encoding of relevant episodes. Participants are likely to incorporate post-encoding misinformation communicated by a social source if they assume that the source is sufficiently trustworthy^16,17^. However, more subtle mechanisms of social appraisal and perceived relevance, operating already at encoding, may likewise contribute to the creation of false memories.

In the present study, we investigated whether false memories can be socially induced in the absence of misinformation. Research on simultaneous co-attention with others^18^, co-representation of information^19^, and distributed task performance^20,21^ suggests that people encode and incorporate to a greater extent information that is relevant to (vs. not relevant to) interaction partners. We argue that people spontaneously monitor current stimuli for relevance to their interaction partner. Stimuli that are relevant to the partner receive more attention, are encoded more deeply, and are thus more readily accessible for further potential co-activities with their partners^18^. As such, information processed by one’s partner is a component, or building block, of shared reality created by interaction partners^22^.

Imagine, for instance, that upon a delivery of goods arriving at a supermarket, Billie and a colleague distribute the task of checking the labels on the unloaded packages. Billie attends to vegetables (e.g., carrots, onions, broccoli); the colleague attends to fruit items (e.g., pears, oranges, grapes). According to our proposal, Billie would monitor the labels not only for vegetable items but also, to some extent, for the partner’s fruit items. This co-monitoring would enhance the cognitive accessibility and recall not only of Billie’s own items but also of the partner’s items. This memory advantage is functional to the extent that it can help to regulate future co-activities and communication, such as organizing product placement and use of shelf space^23^.

Now assume that apples were not delivered. In the absence of the colleague, Billie is later asked by a manager whether apples were received. We propose that, as a result of spontaneous co-processing of the partner’s fruit items, Billie may falsely remember apples as having been delivered. Such false memory is socially rooted to the extent that it emerges from the spontaneous co-processing of the partner’s items. However, it emerges *without misinformation* conveyed by the colleague.

To investigate this subtle, tacit form of false memory, we created a socially contextualized version of the well-known Deese-Roediger-McDermott (DRM) paradigm^24,25^. In individuals, the DRM procedure reliably induces false memories for words that are strongly associated with actually presented word lists. For example, when participants are asked to encode a word list like “table”, “sit”, “seat”, … they subsequently often remember having seen the non-presented lure “chair”. To embed this procedure into a social setting, we presented the lists within a joint encoding paradigm^20^. In this paradigm, two participants simultaneously watch words shown successively on a screen. Each person has to attend and react to a different subset of items (e.g., animal vs. fruit). A third subset of words (e.g., household objects) is assigned to neither participant.

Evidence shows that partner-assigned words (e.g., fruit, in our example) are later better remembered than non-assigned words (household objects), although both categories were not assigned to oneself^20,21^. These results support the notion that participants co-monitored stimuli for partner-relevance, without intentional influence from another person^18,19^. What remains unknown so far, however, is whether the same social co-monitoring mechanism can also induce false memories.

The DRM paradigm is particularly apt to address this question because it reliably creates false memories. These false memories can be even subjectively rich, encompassing vivid memory details^26^. Richness is an important cue for evaluating the truthfulness of reported memories^27,28^. Apart from examining effects on overall memory, we therefore also assessed effects specifically on those memories associated with particularly rich subjective memory experiences.

We performed two experiments that applied the DRM paradigm within adapted versions of the joint encoding paradigm^20^. In Experiment 1, social co-monitoring was explicitly instructed for each stimulus, in Experiment 2 there was no such instruction. To assess the phenomenological richness of memories, we included “Remember/Know” decisions regarding subjective memory experience^29^ in Experiment 1 and asked for source memory as a specific additional memory for encoding context in Experiment 2. These particularly rich memories indicate that participants do not merely think that a specific word had been presented, but even report to still know some additional details or context information from the original presentation of the word at encoding. Both experiments revealed that false memories were augmented for stimuli that were relevant (vs. not relevant) to the partner, and this effect persisted for particularly rich memory experiences.

## Experiment 1

In our first experiment, participants processed the social relevance of each presented stimulus explicitly during the joint encoding task, i.e., they had to decide whether the stimulus was relevant to the partner’s task or not. Because pilot tests produced low false memory rates for non-self-assigned categories in a free recall format, we used a recognition test. Still, following the original DRM procedure^25^, we also included a free recall test preceding the recognition memory test.

### Methods

#### Participants

Fifty-four participants (age: *M* = 24.54 years, *SD* = 6.58; 45 female) were recruited online or personally at the University of Münster (Germany). The sample size was determined on the basis of an a-priori power analysis performed by G*Power^30^ to detect an effect size (*d*_*z*_) of at least 0.5 with high statistical power (power = 0.95; alpha = 0.05, two-tailed). Participants took part in the study for curricular credit or the chance of winning a voucher for an online shop (€10). The study was approved by the ethics committee of the Department of Psychology at the University of Münster and was carried out in accordance with the provisions of the Declaration of Helsinki. All participants gave informed written consent before participation.

#### Materials and procedure

The experimental procedure basically followed the joint encoding procedure as described in Eskenazi et al.^20^, but with some modifications to allow the combination with the DRM paradigm. The primary modification was that not word meaning, but word color determined task relevance of a word. Participants took part in the experiment in pairs. Following the original procedure from Eskenazi et al.^20^, the two participants in a pair performed the first part of the experiment at the same computer, sitting next to each other and using the same keyboard. The study was introduced as a “word processing task with another participant.” Written instructions explained the details of the task. Participants were informed that single words would be presented successively on the computer screen, and that each word could be written in one of the colors blue, green, or red.

Each participant was assigned to one of the three colors and informed that his/her task was to memorize these assigned words. The two participants in each pair were always assigned to two different colors. Both of them knew from the beginning which color was assigned to the other participant and which color was assigned to nobody. The assignment of specific colors to conditions was balanced across participants. Participants were instructed to indicate for each word by a keypress whether it was assigned to themselves, to the partner, or to nobody, and if it was assigned to themselves, to try to memorize it. The pre-defined keys to perform the word categorization were “x”, “c”, and “v” (on the left part of the keyboard) for the person sitting on the left, and “1”, “2”, and “3” on the numlock pad (on the right of the keyboard) for the person sitting on the right.

Instructions emphasized that it was important to memorize the self-assigned words because questions would have to be answered later regarding these self-assigned words. (The expression “memory test” was avoided.) Despite this instructed focus on the self-assigned words, the word categorization task was designed to require a keypress for all stimuli (not only for self-assigned stimuli, as in Eskenazi et al.^20^) in order to ensure participants’ attention across the whole duration of the task. Furthermore, this procedure excluded a confound by perceptual cues due to audible clicks generated by the partner’s keypresses^21^. In the critical comparison between partner-assigned and non-assigned words, such sounds would occur only in the former but not in the latter case, if instructions asked for a keypress only for self-assigned words.

The material used in the joint encoding task consisted of 195 words from 15 German DRM word lists used by Diekelmann et al.^31^. Each list was composed of 13 words (we shortened the original list length of 15 by two words), which were semantically associated with a non-presented theme word, serving as the “critical lure”. For instance, the list for the critical lure *music* contained note, sound, piano, sing, band, melody, guitar, concert, instrument, symphony, jazz, orchestra, and art. Within a list, these words were ordered according to their strength of association with the critical lure. That is, the word in the first position (here: note) had the highest associative strength in relation to the critical lure (here: music), the last position (here: art) had the weakest associative strength to it. (We shortened the original list length in Diekelmann et al.^31^ from 15 to 13, leaving out words from the original positions 5 and 15 of each list.) Importantly, each list was assigned to one of three colors (blue, green, and red) and was presented with a corresponding color. Accordingly, five lists were shown in red, green, and blue color, respectively. In the recognition memory test, additional words not presented at encoding were included. Fifteen of these new words were the critical lures of the previously presented DRM lists. Additional 42 words were distractors (words from the original positions 5 and 15 and twelve completely unrelated words).

E-Prime (Version 2.0) was used to run the experiment. Two practice runs (each containing nine words not included in the main run) were performed to familiarize participants with the task. The main run included all 15 word lists (five for each category), presented listwise in a pseudo-random order in such a way that a word list with a specific task assignment was never directly followed or preceded by another word list with the same task assignment. Additional nine words (three of each color) served as distractors, with three of them presented in the beginning (as primacy buffer) and three in the end (as recency buffer). All words were presented for 2500 ms one after another in the middle of the screen and appeared in their specified color (blue, green, or red) on a black background. Regardless of the response time, the words remained visible for the whole 2500 ms. Participants were instructed in advance to be focused on the task across the whole run and not to talk. Instructions also pointed out that response times did not matter, so that it was not important to do the keypresses for the word categorizations faster than the partner. As in the standard DRM paradigm, lists were presented in blocks, in order to induce the targeted high percentages of false memories for the critical lure that are typically attained with this procedure.

There was no mentioning at encoding of any different importance of other-assigned vs. non-assigned words for the own task. Still, it is possible that participants covertly assumed that partner-assigned words could have more relevance to them than non-assigned words (e.g., by adopting a cooperative or competitive attitude towards the later task that they expected). However, any such subjective assumption would be part of participants’ own tacit assignment of differential importance to social than to non-social stimuli, which we actually aim to investigate here. The full instruction text that participants received can be found as Supplementary Information S1 online.

After having finished the word categorization task, the two participants were separated to perform the subsequent memory tests individually. First, a free recall test was performed, for which each participant received an empty sheet of paper. Unexpectedly, they were asked to write down as many words as possible not only from the self-assigned category in the previous task, but from all three categories. The experimenter repeated this information orally, in order to ensure that participants had understood it. After participants had written down all the words they could recall, the recognition memory test followed on a computer. Instructions emphasized that the memory test again referred to all words presented in the previous word categorization task, regardless of word color. Altogether 132 words, written in white color on a black background, were presented successively in random order. Seventy-five of the words had been actually presented during the word categorization task (words from positions 1, 4, 6, 13, and 14 of the original 15-item word lists from Diekelmann et al.^31^). Additional 57 words were new. Fifteen of these new words were the critical lures of the previously presented DRM lists, which are in the focus of interest here.

The remaining new words were distractors (30 words from the original list positions 5 and 15 and twelve additional unrelated words). For each word, participants had to indicate whether it had been presented during the word categorization task or not, and if yes, whether their answer was based on a “remember” or “know” experience^29^. They were instructed to give a “remember” answer if they were able to retrieve additional details or context information from the respective encoding situation, and to give a “know” answer if this was not the case (see Supplementary Information S2 online, for the exact explanation that participants received regarding the distinction between “remember” and “know” experiences in memory). Participants completed the task at their own pace.

#### Statistical analyses

Performance rates in the free recall test were too low in this modified task paradigm in the non-self-assigned word categories “Other” and “None” (overall < 5%) to allow statistical analyses. Many participants did not even mention one word in these conditions. Mean recall values (absolute numbers and proportions) were for veridical memories: Self = 17.63 items (as proportion of 65 possible items: 0.27), Other = 2.01 items (0.03), None = 1.28 items (0.02); and for false memories: Self = 1.37 items (as proportion of 5 possible items: 0.27), Other = 0.25 items (0.05), None = 0.17 items (0.03).

Dependent variables in the recognition memory test were the number of “Yes” answers to the question whether the presented item was shown during the word categorization task or not (overall memory). For an additional analysis on only memories with the richest subjective memory experiences, only “Yes” answers with additional indication of a “remember” experience were considered. For false memories, proportions of respective answers were calculated with regard to the five critical lures in each word category (self, other, none), and for veridical memory with regard to the 25 list words shown at recognition testing that had actually been presented at encoding. For control purposes, we also calculated the proportion of “Yes” answers to the 42 distractor items as a critical indicator of a general “yes” response bias (baseline false alarm rate). This baseline value was used to determine the discrimination parameter *P*_r_^32^, both for veridical memories (*P*_r_ = hit rate [*HR*] minus false alarm rate for distractors [*FA*-D]) and false memories (*P*_r_ = false alarm rate for critical lures [*FA*-CL] minus false alarm rate for distractors [*FA*-D]). A positive value of *P*_r_ that differs significantly from zero indicates “real” memory beyond chance level.

All statistical analyses were performed in R^33^, and we used the lme4 package for multilevel analyses^34^. Using this approach to analyze our recognition memory data, we aggregated the number of “Yes” answers to the question whether the presented item was shown during the word categorization task or not across all participants, and treated this proportion as dependent variable. Given that each participant had 132 trials (presented words) it is likely that participants had different intercepts. Therefore, we treated the individual trials as nested within participants to account for the variation around the intercepts for participants^35^. We also introduced a random intercept for the words that were shown.

Data were converted to a long thin data format with 7128 lines in the data matrix (54 participants × 132 words). The dependent variable in the recognition memory test was how the participant responded (Yes/No) to each presented words in the list. Due to the dichotomous nature of the responses, we used a multilevel logistic model^35,36^. The independent variable was our within-subject factor word category (self, other, none). We first tested a model with the independent variable against a random intercept only model to gauge whether the factor word category significantly contributed to improved model fit in a ${\upchi }^{2}$-likelihood ratio test. Using dummy coding, the “none” category constituted the reference group.

We conducted our analyses separately for veridical memories and false memories. For both analyses (veridical and false memories), an additional analysis was conducted on memories with the richest subjective memory experiences, that is, only “Yes” answers with additional indication of a “remember” experience. Based on previous findings, proportions for self-assigned words were expected to be generally higher than those in the corresponding “other” and “none” categories. However, the critical statistical test in all analyses was the pairwise comparison between the two categories “other” and “none”. To facilitate interpretation, we also report the odds ratio (OR) alongside the logit (β) estimates.

We used a multilevel approach for our statistical analyses, because the assumptions on which conventional analyses of variance (ANOVAs) are based were not fully met (in particular regarding normal distribution and sphericity). The multilevel approach is better suited than ANOVAs to deal with violations of different assumptions regarding specific data properties^37^. However, we note that when ANOVAs were still performed instead of multilevel analyses, all statistical results were essentially the same.

All original data and R codes are available online at: https://osf.io/mukfn/?view_only=2bce4b0c2a2041cab14ec528fd6b526a.

### Results

Recognition memory data are shown in Table 1, for both veridical and false memories. In all cases, the overall discrimination parameter *P*_r_ was significantly higher than zero (all *p*s ˂ 0.001), indicating genuine veridical and false memories beyond a simple response bias (means and 95% CI for veridical overall memory: *HR* = 0.50 [0.49; 0.52], *FA*-D = 0.22 [0.21; 0.24], *P*_r_ = 0.28 [0.24; 0.32]; for veridical “remember” memory: *HR* = 0.30 [0.28; 0.31], *FA*-D = 0.07 [0.06; 0.08], *P*_r_ = 0.23 [0.19; 0.27]; for false overall memory *FA*-CL = 0.55 [0.52; 0.58], *FA*-D = 0.22 [0.21; 0.24], *P*_r_ = 0.33 [0.28; 0.38]; for false “remember” memory *FA*-CL = 0.30 [0.27; 0.33], *FA*-D = 0.07 [0.06; 0.08], *P*_r_ = 0.23 [0.18; 0.28]).

**Table 1:** Veridical and false memories in Experiment 1.

Category	Overall memory				“Remember” memory			
	Veridical memories		False memories		Veridical memories		False memories	
	*M*	*SD*	*M*	*SD*	*M*	*SD*	*M*	*SD*
Self	0.78	0.41	0.80	0.40	0.59	0.49	0.57	0.50
Other	0.40**	0.49	0.48**	0.50	**0.15**	0.35	0.21**	0.41
None	**0.33**	0.47	**0.36**	0.48	**0.14**	0.36	**0.12**	0.33

Data indicate mean proportions (*M*) and standard deviations (*SD*) for all "Yes" answers in the recognition memory test (left panel) and for only those "Yes" answers with additional indication of a “remember” experience (right panel), shown for actually presented words (veridical memories) and for critical lures (false memories) separately for the three word categories (Self, Other, None). Proportion values refer to 25 items for veridical memories and to 5 items for false memories (critical lures). All “Self” means differ significantly from the corresponding means in the other two categories (all *p*s < 0.001). Critical statistical comparisons (shown in bold) refer to differences in the means between the two categories “Other” and “None”, in which words were not relevant to the own task and therefore only incidentally encoded. **p < 0.01, for difference between “Other” and “None”.

For both overall veridical memory, likelihood ratio, ${\upchi }^{2}$(2) = 761, *p* ˂ 0.001, and veridical “remember” memory, likelihood ratio, ${\upchi }^{2}$(2) = 1005, *p* ˂ 0.001, adding the factor category led to a significant model improvement. In both cases, the numbers were substantially higher for self-assigned words than for words from the other two categories (all *p*s < 0.001). There were also substantially more veridical memories in the other-assigned than in the non-assigned word category for overall memory, *β* = 0.36, OR = 1.44 [CI 95% 1.21; 1.71], *SE* = 0.08, *z* = 4.14, *p* < 0.001, but not for specifically „remember“-based memory, *p* = 0.166.

For false memories, adding the factor category led to model improvement for both overall, likelihood ratio, ${\upchi }^{2}$(2) = 139, *p* ˂ 0.001, and “remember” only responses, likelihood ratio, ${\upchi }^{2}$(2) = 188, *p* ˂ 0.001. False memories were substantially higher for self-assigned words than for words from the other two categories (all *p*s < 0.001). Critically, there were also significantly more false memories in the other-assigned than in the non-assigned word category both in the overall memory analysis, *β* = 0.60, OR = 1.82 [CI 95% 1.24; 2.67], *SE* = 0.19, *z* = 3.09, *p* = 0.002 and when only “remember” answers were taken into account, *β* = 0.86, OR = 2.63 [CI 95% 1.37; 4.06], *SE* = 0.27, *z* = 3.11, *p* = 0.002.

It is to be noted that our 42 distractor items encompass two different types of items, i.e., the 30 words that belonged to positions 5 and 15 of the original DRM lists, but were not shown at encoding, and the 12 words that were not part of any of the lists. Hence, the items from list positions 5 and 15 can be regarded as intermediate items: they do not represent the semantic “culmination point” of the respective list (common theme word to all words in a list), as the critical lure does, but they still have some semantic association to the list words that were actually presented. This means that they could be also seen as a subtype of “critical lures” for the shown lists, because they also have some semantic association with them. In this view, only the 12 list-independent items would actually serve as distractors. Following this rationale, we also performed alternative analyses, in which the words from list positions 0 (i.e., critical lures in the strict sense) were combined with the words from positions 5 and 15 to define a broader word category of “critical lures”. In all cases, these alternative analyses yielded the same statistical results as the original analyses. Results of all these alternative analyses can be found as Supplementary Information S4 online.

## Experiment 2

Experiment 2 replicated and extended Experiment 1. Importantly, we tested if the effect would persist in the absence of explicit instructions to process the social meaning of stimuli during the joint-encoding task. For this purpose, participants were not asked to determine word assignment (self, other, or none) in the categorization task, but to indicate the word color (blue, green, or red). Thus, participants had to infer the differential social meaning of the non-self-stimuli on their own. If the effect persists under these conditions, this would further support the notion that the underlying process of social appraisal occurs spontaneously^18,19^. In addition, two procedural changes were introduced in Experiment 2. First, the initial free recall test was omitted. Second, we used another method to assess particularly rich memories. For each word that participants recognized as having been presented, they were not asked for their “remember” vs. “know” experience, but to indicate to which of the three word categories the recognized word belonged (source memory).

### Methods

#### Participants

Sixty participants (age: *M* = 24.91 years, *SD* = 3.84; 42 female) were recruited online or personally at the University of Münster. Participants took part in the study for curricular credit or the chance of winning a voucher for an online shop (€10). All participants gave informed written consent before participation. One participant was excluded from statistical analyses due to an extremely liberal response bias, as indicated by a percentage of “Yes” answers > 95% even for the distractors in the recognition memory test that were not critical lures.

#### Materials and procedure

Material and procedures were identical to Experiment 1, with the following exceptions: first, during the word categorization task, participants were asked to determine the color of the presented word (not the word assignment). i.e., whether it was written in blue, green, or red color (see Supplementary Information S3 online, for full text of modified instructions). Second, there was no free recall test, but only a recognition memory test. Third, in the recognition test, participants were specifically asked for their source memory, as an alternative to the “remember/know” distinction to define rich memories. Specifically, for “Yes” answer in the recognition test, participants were not asked to report their subjective experience of “remembering” vs. “knowing.” Instead, they indicated the word category of the recognized word, i.e., the word color, indicative of task assignment (self, other, none) and therefore also social relevance at encoding. Thus, regarding false memories, any “Yes” answer to a critical lure, where participants additionally chose as remembered color the color of the list to which this (actually not presented) item belonged, counted as a false memory with additional (false) source memory, representing a particularly rich false memory. As in Experiment 1, recognition memory analyses were based on a multilevel approach, using a long thin data format (here 7788 lines, resulting from 59 participants × 132 words).

### Results

Data for the recognition memory test (Table 2) replicate the findings from Experiment 1. For both veridical and false memories, the overall discrimination parameter *P*_r_ was significantly higher than zero (all *p*s ˂ 0.001), indicating genuine memories beyond a simple response bias (means and 95% CI for veridical overall memory: *HR* = 0.61 [0.59; 0.62], *FA*-D = 0.33 [0.31; 0.35], *P*_r_ = 0.28 [0.24; 0.32]; for veridical source memory: *HR* = 0.46 [0.44; 0.47], *FA*-D = 0.18 [0.16; 0.19], *P*_r_ = 0.28 [0.24; 0.32]; for false overall memory *FA*-CL = 0.64 [0.61; 0.67], *FA*-D = 0.33 [0.31; 0.35], *P*_r_ = 0.31 [0.26; 0.36]; for false source memory *FA*-CL = 0.50 [0.47; 0.53], *FA*-D = 0.18 [0.16; 0.19], *P*_r_ = 0.32 [0.28; 0.36]).

**Table 2:** Veridical and false memories in Experiment 2.

Category	Overall memory				Source memory			
	Veridical memories		False memories		Veridical memories		False memories	
	*M*	*SD*	*M*	*SD*	*M*	*SD*	*M*	*SD*
Self	0.83	0.38	0.84	0.37	0.71	0.45	0.77	0.42
Other	0.52**	0.50	0.58*	0.49	0.35**	0.48	0.42**	0.49
None	**0.47**	0.50	**0.49**	0.37	**0.31**	0.46	**0.31**	0.46

Data indicate mean proportions (*M*) and standard deviations (*SD*) for all "Yes" answers in the recognition memory test (left panel) and for only those "Yes" answers with additional indication of respective source memory (right panel), shown for actually presented words (veridical memories) and for critical lures (false memories) separately for the three word categories (Self, Other, None). Proportion values refer to 25 items for veridical memories and to 5 items for false memories (critical lures). All “Self” means differ significantly from the corresponding means in the other two categories (all *p*s < 0.001). Critical statistical comparisons (shown in bold) refer to differences in the means between the two categories “Other” and “None”, in which words were not relevant to the own task and therefore only incidentally encoded. *p < 0.05, **p < 0.01, for difference between “Other” and “None”.

For both overall veridical memory, likelihood ratio, ${\upchi }^{2}$(2) = 573, *p* ˂ 0.001, and veridical memories with additional corresponding source memory, likelihood ratio, ${\upchi }^{2}$(2) = 680, *p* ˂ 0.001, the factor category contributed to significant model improvement. The numbers were always significantly higher for self-assigned words than for words from the other two categories (all *p*s < 0.001). There were also more veridical memories in the other-assigned than in the non-assigned word category for overall memory, *β* = 0.27, OR = 1.31 [CI 95% 1.12; 1.54], *SE* = 0.08, *z* = 3.32, *p* < 0.001, and also for memories with additional source memory, *β* = 0.24, OR = 1.26 [CI 95% 1.07; 1.49], *SE* = 0.08, *z* = 2.91, *p* = 0.005.

Regarding false memories, adding the factor category likewise led to model improvement for both overall false memories, likelihood ratio, ${\upchi }^{2}$(2) = 99, *p* ˂ 0.001, and for false memories with additional source memory, likelihood ratio, ${\upchi }^{2}$(2) = 160, *p* ˂ 0.001. False memories were higher for self-assigned words than for words from the other two categories (all *p*s < 0.001). Critically, there were again also significantly more false memories in the other-assigned than in the non-assigned word category both in the overall memory analysis, *β* = 0.42, OR = 1.52 [CI 95% 1.07; 2.16], *SE* = 0.18, *z* = 2.34, *p* = 0.019, and when only memories with additional source memory were taken into account, *β* = 0.55, OR = 1.74 [CI 95% 1.21; 2.49], *SE* = 0.18, *z* = 3.00, *p* = 0.003.

As in Experiment 1, alternative analyses, in which the words from list positions 5 and 15 were additionally defined as “critical lures”, yielded the same statistical results as the original analyses (see Supplementary Information S4 online).

## General discussion

The present study reveals a novel type of socially driven false memories, which emerges from co-processing during initial encoding. In contrast to previous demonstrations, this effect appears to emerge unobtrusively from the spontaneous monitoring of socially relevant stimuli at encoding rather than by post-encoding presentation of misinformation leading to conflicting memory representations. Using the DRM false memory task^25^ within the social context of a distributed task sharing paradigm^20^, we replicated previous findings that the mere knowledge that words were specifically relevant to a partner’s task led to enhanced memory for these words^20,21^. Critically, however, false memories for words that were semantically highly associated with the actually presented partner-relevant words were also significantly enhanced. As shown in Experiment 2, this was also the case when instructions did not directly demand processing of social relevance vs. irrelevance of each stimulus during task performance (as in Experiment 1). This is in line with the assumption of naturally occurring involuntary social appraisal as the underlying mechanism^18,19^.

Although the demonstrated social process of false memory formation is new, the basic cognitive mechanism that it affects is not. It is the same mechanism of associative semantic inference already observed in many previous studies performed with individual participants, where DRM lists inherently only have self-relevance^26^. This basic effect was clearly confirmed here for the self-assigned words that showed in all analyses the strongest extent of false memory generation (in parallel to the best veridical memory for actually presented words from this word category). The strongest effect for the self-assigned words is not surprising because participants had to focus their attention to these words already by instruction, just as in classical DRM studies.

Critically, the present data show, for the first time, that the same cognitive mechanism is effective, albeit in an attenuated manner, when attention to stimuli is solely driven by attribution of social relevance. Participants developed more false memories for word lists that were relevant to the partner than for word lists that were not, although both types of word lists were equally irrelevant to perform the own task and were therefore only encoded incidentally. Compared to paradigms relying on post-event misinformation^6–8^, social influence here is very subtle, being exerted only passively by an interaction partner. Nevertheless, a remarkable feature of the effects in the present experiments is that the false memories that they create are relatively rich and vivid, accompanied by additional memories for detail or context. Notably, when only these memories with high subjective richness were considered, the same statistical pattern was obtained.

False memories from interpersonal co-monitoring at encoding also differ from other paradigms that describe negative effects of social processes on memory performance, such as “collaborative inhibition”^3^ and “socially shared retrieval-induced forgetting”^38^. These phenomena investigate the extent of forgetting of originally encoded material, rather than the active creation of false memories, i.e., of non-veridical new memories. Furthermore, like misinformation studies, they are not interested in social influence at encoding, but on effects of social processes after encoding. This latter aspect also distinguishes the present effects from false memories of action performance from the mere observation of others’ actions^39^, and from effects of collaborative recall of DRM lists on the transmission of false memories^40^.

The present findings not only advance our theoretical understanding of how social and cognitive factors interact in false memory formation. Because episodic memory also affects the quality of judgment and decision-making^13,41^, this understanding is also relevant to applied research, e.g., in the context of economic and consumer choices^12,42^. Particularly obvious is the pertinence to the forensic domain, considering the fact that a substantial proportion of false legal convictions is based on false eyewitness memories^14,43^. Notably, research on social factors in false memory generation has so far mainly focused on interpersonal communication in active social interaction taking place after encoding. Our results show that it is likewise important to consider social determinants that exert their influence more unobtrusively already at encoding. This does not mean that post-encoding effects cannot be relatively subtle as well. For example, Harris et al.^9^ found that simply hearing a confederate tell an own autobiographical memory narrative influenced participants' own memory reports of a similar autobiographical event (e.g., with both narratives referring to the own final high school exam). However, social influence in this case is still based on information openly presented by another person, which is then erroneously incorporated into an own memory account. Furthermore, these memories are not necessarily false memories, because participants might predominantly incorporate information from the partner that was also true of their own respective autobiographical experience (e.g., that the hand was sore from writing after the exam).

One limitation of the present study is the relatively low number of items that served to estimate the false memory rates (five items per condition). However, we obtained the same pattern of results also in alternative statistical analyses where distractors with moderate semantic relation to the previously presented word lists were included in the definition of critical lures, so that here were 15 rather than only 5 critical lures in each condition. Still, it would be desirable to replicate our findings with more DRM lists per condition, although using more lists would necessarily also increase task duration.

In sum, our data show for the first time that social induction of false memories, notably even subjectively rich ones, does not necessarily require communication of deceptive information. Rather than acting through post-encoding open communication, the mechanism of co-monitoring that we describe here is driven by socially guided attention at encoding. Because social guidance of attention is ubiquitous in human life^1,18,19^, this potential source of even rich false memories deserves deeper consideration in future research, including applied research (e.g., regarding the evaluation of eyewitness memories).

## Supplementary Information

Supplementary Information.
