# Supplementary material for: Socially induced false memories in the absence of misinformation
Source: Sci Rep. 2022 May 11;12:7725. doi: 10.1038/s41598-022-11749-w (PMC9095591; doi:10.1038/s41598-022-11749-w)
Supplement: Supplementary file 1 — Supplementary Information. [file 41598_2022_11749_MOESM1_ESM.docx]

**Supplementary Information**

for the article

**“Socially induced false memories in the absence of misinformation”**

Ullrich Wagner^1^*, Pascal Schlechter^2^ & Gerald Echterhoff^1^

^1^Department of Psychology, University of Münster, Germany

^2^Department of Psychiatry, University of Cambridge, United Kingdom

**Supplementary Information S1:**

**Instructions Experiment 1**

**Instructions Experiment 1 - Original German version:**

(Example of altogether six different versions regarding specific color assignments)

Liebe Versuchsperson,

sie führen jetzt zu zweit gemeinsam eine Aufgabe zur Verarbeitung von Wörtern durch. Die Aufgabe ist ganz einfach: Jeder von Ihnen beiden muss sich Wörter einer bestimmten Farbe einprägen, wobei jeder von Ihnen auf eine andere Farbe achten muss.

**Es werden viele verschiedene Wörter nacheinander präsentiert. Jedes Wort kann eine von drei Farben haben, nämlich blau, rot, oder grün.**

**Der *links* vor dem Computer sitzenden Person ist die Farbe blau zugeordnet, d.h ihre Aufgabe besteht darin, sich speziell die blauen Wörter einzuprägen.**

**Der *rechts* vor dem Computer sitzenden Person ist die Farbe rot zugeordnet, d.h ihre Aufgabe besteht darin, sich speziell die roten Wörter einzuprägen.**

Für Sie beide ist es wichtig, sich jeweils die Wörter der eigenen Farbe gut einzuprägen, da Sie nachher Fragen zu diesen Ihnen zugeordneten Wörtern beantworten werden.

Es kann in manchen Versionen des Programms vorkommen, dass über längere Zeit kein Wort in der Ihnen zugeordneten Farbe erscheint. Es ist daher wichtig, dass Sie über die gesamte Zeit der Aufgabe immer voll konzentriert bleiben, denn Wörter Ihrer Farbe können trotzdem jederzeit erscheinen.

**Um zu prüfen, dass Sie beide über die gesamte Zeit der Aufgabe wirklich konzentriert sind, wird jeder von Ihnen beiden bei jedem Wort zunächst per Tastendruck angeben, zu welcher Wortkategorie das Wort gehört: zu der eigenen Wortkategorie, zu der Wortkategorie der anderen Person, oder zu der dritten Kategorie, die niemandem zugeordnet ist.**

Wenn Sie links am Computer sitzen, benutzen Sie für diese Antworten bitte mit der rechten Hand die Tasten „x“, „c“ und „v“: Zeigefinger („x“) für die eigene Wortkategorie, Mittelfinger („c“) für die Wortkategorie des Partners, Ringfinger („v“) für die dritte Wortkategorie, die niemandem zugeordnet ist.

Wenn Sie rechts am Computer sitzen, benutzen Sie für diese Antworten bitte mit der rechten Hand die Tasten „1“, „2“ und „3“ auf dem Num Pad: Zeigefinger („1“) für die eigene Wortkategorie, Mittelfinger („2“) für die Wortkategorie des Partners, Ringfinger („3“) für die dritte Wortkategorie, die niemandem zugeordnet ist.

**Zusammenfassend gibt es also drei verschiedene Ereignisse:**

1. **Ein blaues Wort erscheint:**

- **Wenn Sie *links* sitzen, bestätigen Sie per Tastendruck, dass dieses Wort Ihnen zum Einprägen zugeordnet ist und prägen sich das Wort ein.**
- **Wenn Sie *rechts* sitzen, bestätigen Sie per Tastendruck, dass dieses Wort der anderen Person zugeordnet ist.**

1. **Ein rotes Wort erscheint:**

- **Wenn Sie *rechts* sitzen, bestätigen Sie per Tastendruck, dass dieses Wort Ihnen zum Einprägen zugeordnet ist und prägen sich das Wort ein.**
- **Wenn Sie *links* sitzen, bestätigen Sie per Tastendruck, dass dieses Wort der anderen Person zugeordnet ist.**

1. **Ein grünes Wort erscheint:**

- **Wenn Sie *links oder rechts* sitzen, bestätigen Sie per Tastendruck, dass dieses Wort niemandem von Ihnen beiden zugeordnet ist.**

Wie erwähnt, dient Tastendruck zu jedem Wort nur zur Prüfung Ihrer Aufmerksamkeit über die gesamte Zeit. Es ist dabei wichtiger, dass Sie möglichst immer korrekt antworten, als dass Sie schnell antworten. Es ist auch nicht wichtig, wer von Ihnen beiden schneller antwortet. Am wichtigsten ist es, bei den Antworten möglichst wenige Fehler zu machen, denn dies spricht für eine optimale Aufmerksamkeit. Wenn Ihnen trotzdem mal ein einzelner Fehler unterlaufen sollte (falsche Taste oder gar kein Tastendruck bei einem Wort), ist es nicht so schlimm. Arbeiten Sie dann einfach trotzdem konzentriert weiter und versuchen Sie, weitere Fehler zu vermeiden.

Hinweis: Die Wörter bleiben auch nach einem Tastendruck immer eine gewisse weitere Zeit auf dem Bildschirm, d.h. ein Tastendruck führt nicht dazu, dass das Wort verschwindet. Dies ist absichtlich so und bedeutet nicht, dass die Tastendrücke nicht registriert wurden.

Der Versuchsleiter wird jetzt mit Ihnen beiden zunächst den *Probedurchgang* am Computer durchführen, damit Sie schon vor dem Start des echten Programms mit dem generellen Versuchsablauf dieses Durchgangs in der Praxis vertraut sind.

Wenn Sie jetzt noch Fragen zum Ablauf haben, wenden Sie sich bitte an den Versuchsleiter.

**Instructions Experiment 1 - English translation:**

(Example of altogether six different versions regarding specific color assignments)

Dear participant,

you are now going to perform a word processing task together in pairs. The task is quite simple: each of you two has to memorize words of a certain color, with each of you paying attention to a different color.

**Many different words will be presented one after another. Each word can be shown in one of three colors: blue, red, or green.**

**The person sitting on the *left* at the computer is assigned to the color blue, i.e. their task is to memorize specifically the blue words.**

**The person sitting on the *right* at the computer is assigned to the color red, i.e. their task is to memorize specifically the red words.**

For both of you, it is important to memorize the words of the own assigned color because you will later have to answer questions about these self-assigned words.

In some versions of the program, it can happen that for a long time no word appears in the color assigned to yourself. It is therefore important that you remain fully concentrated throughout the task, as words of your color may still appear at any time.

**To check that both of you are really concentrated for the entire duration of the task, each of you two will first indicate by a keypress for each word to which category the word belongs: to the own word category, to the other person's word category, or to the third category, which is not assigned to anyone.**

If you are sitting on the left side of the computer, please use the keys "x", "c" and "v" with your right hand for these answers: Index finger ("x") for your own word category, middle finger ("c") for your partner's word category, ring finger ("v") for the third word category that is not assigned to anyone.

If you are sitting on the right side of the computer, please use the keys "1", "2" and "3" on the Num Pad with your right hand for these answers: index finger ("1") for your own word category, middle finger ("2") for your partner's word category, ring finger ("3") for the third word category that is not assigned to anyone.

**In summary, there are three different events:**

**1) A blue word appears:**

**- If you are sitting on the *left*, confirm by keypress that this word is assigned to you and memorize the word.**

**- If you are sitting on the *right*, confirm by keypress that this word is assigned to the other person.**

**2) A red word appears:**

**- If you are sitting on the right, confirm by keypress that this word is assigned to you and memorize the word.**

**- If you are sitting on the left, confirm by keypress that this word is assigned to the other person.**

**3) A *green word* appears:**

**- If you are sitting on the *left or right*, confirm by keypress that this word is not assigned to either of you.**

As mentioned, the keypresses to each word only serve to check your attention over time. It is more important that you always answer correctly, if possible, than that you answer quickly. It is also not important who of you answers faster. The most important point is to make as few mistakes as possible in your answers because this indicates optimal attention. If you still make a single mistake (wrong keypress or no keypress at all for a word), it is not consequential. Just continue to work in a concentrated manner and try to avoid further mistakes.

Note: The words will always remain on the screen for a certain additional amount of time after a keypress, i.e. a keypress does not cause the word to disappear. This is by design and does not mean that the keypresses were not registered.

The experimenter will now initially perform a practice run on the computer with both of you so that you are already familiar with the general experimental procedures when the actual run starts.

If you now have any questions about the procedure, please contact the experimenter.

**Supplementary Information S2:**

**Instructions regarding the distinction between „Remember“ and „Know“ experiences in memory**

**Explanation „remember“ vs. „know“ - Original German version:**

**„Erinnert“-Antwort (E)**

Wenn das Gedächtnis für ein Wort von einem bewussten, lebhaften Erinnern begleitet ist, dann gilt das Wort als „erinnert“. „Erinnern“ ist die Fähigkeit, sich bewusst zu werden, was Sie erlebten oder was Ihnen aufgefallen ist, als das Wort präsentiert wurde. Dies können bestimmte Aspekte des Wortbildes sein oder etwas, das in dem Raum passierte (z.B., ein Fenster schlug zu) oder an was man gerade gedacht hat, bzw. was man gerade tat, während das Wort präsent war. Mit anderen Worten, das „erinnerte“ Wort sollte eine lebhafte Assoziation in Erinnerung rufen, ein Bild oder etwas Persönliches zu der Zeit der Wortpräsentation oder auch etwas über die Erscheinung des Wortes oder dessen Position (bspw. was kam vor oder nach dem Wort?).

**„Gewusst“-Antwort (G)**

„Gewusst“-Antworten sollten dann gegeben werden, wenn Sie zwar meinen, dass das Wort vorkam, Sie sich aber nicht bewusst an seine vorige Präsenz erinnern bzw. nichts erinnern können, was mit dem Vorkommen des Wortes verbunden ist. Geben Sie die Antwort „Gewusst“, wenn Sie meinen, dass das Wort vorkam, es Ihnen aber nicht gelingt, spezifische Erinnerungen an die Wortpräsentation hervorzurufen.

Beispiel zur Unterscheidung zwischen „erinnert“- und „gewusst“-Antworten:

Wenn man an der Bushaltestelle steht und jemanden sieht, fällt einem manchmal auf, dass man diese Person schon einmal gesehen hat. Jetzt ist es möglich, dass man sich erinnert, dass man diese Person letzten Freitag, in einem lila Regenmantel an der gleichen Bushaltestelle gesehen hat. Man kann sich also an dazugehörige Details erinnern. Unter diesen Umständen sprechen wir von „erinnern“. Es wäre aber auch möglich, dass man sich zwar im Klaren darüber ist, dass man diese Person schon einmal gesehen hat, sich aber nicht mehr daran erinnern kann, wann und wo man sie gesehen hat, oder was sie getragen hat. Man erinnert also keine dazugehörigen Details. Man weiß aber wohl, dass man diese Person schon einmal gesehen hat. Unter diesen Umständen sprechen wir von „gewusst“.

**Explanation „remember“ vs. „know“ – English translation:**

**"Remember" response (R)**

If your memory for a word is accompanied by a conscious, vivid recollection, the word counts as “remembered”. "Remembering" is the ability to become aware of what you experienced or what you noticed when the word was presented. This may be certain visual aspects of the word or something that happened in the room (e.g., a window slammed) or what you were thinking about or doing while the word was present. In other words, the "remembered" word should evoke a vivid association, an image or something personal at the time the word was presented, or something about the appearance of the word or its position (e.g., what came before or after the word?).

**"Know" response (K)**

"Know" responses should be given when you think the word occurred, but you do not consciously remember its previous presence or cannot recall anything associated with the word's occurrence. Answer "know" if you think the word occurred but you fail to evoke specific memories of the word's presentation.

An example to distinguish between "remember" and "know" responses:

When you are standing at the bus stop and see someone, you sometimes notice that you have seen that person before. You may remember that you saw this person last Friday, wearing a purple raincoat at the same bus stop. Thus, you can remember associated details. Under these circumstances, we speak of a "remember" experience. However, it is also be possible that you are aware that you have seen this person before, but cannot remember when and where you saw the person, or what the person was wearing. Thus, you do not remember any associated details. However, you still know that you have seen this person before. Under these circumstances, we speak of a "know" experience.

**Supplementary Information S3:**

**Instructions Experiment 2**

**Instructions Experiment 2 - Original German version:**

(Example of altogether six different versions regarding specific color assignments)

Liebe Versuchsperson,

Sie führen jetzt zu zweit gemeinsam eine Aufgabe zur Verarbeitung von Wörtern durch. Die Aufgabe ist ganz einfach: Jeder von Ihnen beiden muss sich Wörter einer bestimmten Farbe einprägen, wobei jeder von Ihnen auf eine andere Farbe achten muss.

**Es werden viele verschiedene Wörter nacheinander präsentiert. Jedes Wort kann eine von drei Farben haben, nämlich blau, grün oder rot.**

**Der *links* vor dem Computer sitzenden Person ist die Farbe blau zugeordnet, d.h. ihre Aufgabe besteht darin, sich speziell die blauen Wörter einzuprägen.**

**Der *rechts* vor dem Computer sitzenden Person ist die Farbe rot zugeordnet, d.h. ihre Aufgabe besteht darin, sich speziell die roten Wörter einzuprägen.**

Für Sie beide ist es wichtig, sich jeweils die Wörter der eigenen Farbe gut einzuprägen, da Sie nachher Fragen zu diesen Ihnen zugeordneten Wörtern beantworten werden.

Es kann in manchen Versionen des Programms vorkommen, dass über längere Zeit kein Wort in der Ihnen zugeordneten Farbe erscheint. Es ist daher wichtig, dass Sie über die gesamte Zeit der Aufgabe immer voll konzentriert bleiben, denn Wörter Ihrer Farbe können trotzdem jederzeit erscheinen.

**Um zu überprüfen, dass Sie beide über die gesamte Zeit der Aufgabe wirklich konzentriert sind, wird jeder von Ihnen beiden bei jedem Wort zunächst per Tastendruck angeben, zu welcher Farbkategorie das Wort gehört**, wobei Sie für die Antworten bitte folgendermaßen Zeigefinger, Mittelfinger und Ringfinger der rechten Hand benutzen:

**Zeigefinger = blau (= Farbkategorie, die der links sitzenden Person zugeordnet ist)**

**Mittelfinger = grün (= Farbkategorie, die niemandem zugeordnet ist)**

**Ringfinger = rot (= Farbkategorie, die der rechts sitzenden Person zugeordnet ist)**

Wenn Sie rechts am Computer sitzen, benutzen Sie für diese Antworten bitte mit der rechten Hand die Tasten „1“, „2“ und „3“ auf dem Num Pad: Zeigefinger („1“) für blau, Mittelfinger („2“) für grün, und Ringfinger („3“) für rot.

Wenn Sie links am Computer sitzen, benutzen Sie für diese Antworten bitte mit der rechten Hand die Tasten „x“, „c“ und „v“: Zeigefinger („x“) für blau, Mittelfinger („c“) für grün, und Ringfinger („v“) für rot.

**Zusammenfassend gibt es also drei verschiedene Ereignisse:**

1. **Ein blaues Wort erscheint:**

- **Wenn Sie *links* sitzen, bestätigen Sie per Tastendruck mit dem Zeigefinger, dass dieses Wort blau ist und prägen sich das Wort ein.**
- **Wenn Sie *rechts* sitzen, bestätigen Sie per Tastendruck mit dem Zeigefinger, dass dieses Wort blau ist.**

1. **Ein grünes Wort erscheint:**

- **Egal, ob Sie links oder rechts sitzen: Sie bestätigen per Tastendruck mit dem Mittelfinger, dass dieses Wort grün ist.**

1. **Ein rotes Wort erscheint:**

- **Wenn Sie *rechts* sitzen, bestätigen Sie per Tastendruck mit dem Ringfinger, dass dieses Wort rot ist und prägen sich das Wort ein.**
- **Wenn Sie *links* sitzen, bestätigen Sie per Tastendruck mit dem Ringfinger, dass dieses Wort rot ist.**

Wie erwähnt, dient Tastendruck zu jedem Wort nur zur Prüfung Ihrer Aufmerksamkeit über die gesamte Zeit. Es ist dabei wichtiger, dass Sie möglichst immer korrekt antworten, als dass Sie schnell antworten. Es ist auch nicht wichtig, wer von Ihnen beiden schneller antwortet. Am wichtigsten ist es, bei den Antworten möglichst wenige Fehler zu machen, denn dies spricht für eine optimale Aufmerksamkeit. Wenn Ihnen trotzdem mal ein einzelner Fehler unterlaufen sollte (falsche Taste oder gar kein Tastendruck bei einem Wort), ist es nicht so schlimm. Arbeiten Sie dann einfach trotzdem konzentriert weiter und versuchen Sie, weitere Fehler zu vermeiden.

Hinweis: Die Wörter bleiben auch nach einem Tastendruck immer eine gewisse weitere Zeit auf dem Bildschirm, d.h. ein Tastendruck führt nicht dazu, dass das Wort verschwindet. Dies ist absichtlich so und bedeutet nicht, dass die Tastendrücke nicht registriert wurden.

Der Versuchsleiter / die Versuchsleiterin wird jetzt mit Ihnen beiden zunächst den *Probedurchgang* am Computer durchführen, damit Sie schon vor dem Start des echten Programms mit dem generellen Versuchsablauf dieses Durchgangs in der Praxis vertraut sind.

Wenn Sie jetzt noch Fragen zum Ablauf haben, wenden Sie sich bitte an den Versuchsleiter / die Versuchsleiterin.

**Instructions Experiment 2 - English translation:**

(Example of altogether six different versions regarding specific color assignments)

Dear participant,

you are now going to perform a word processing task together in pairs. The task is quite simple: each of you two has to memorize words of a certain color, with each of you paying attention to a different color.

**Many different words will be presented one after another. Each word can be shown in one of three colors: blue, green, or red.**

**The person sitting on the *left* at the computer is assigned to the color blue, i.e. their task is to memorize specifically the blue words.**

**The person sitting on the *right* at the computer is assigned to the color red, i.e. their task is to memorize specifically the red words.**

For both of you, it is important to memorize the words of the own assigned color because you will later have to answer questions about these self-assigned words.

In some versions of the program, it can happen that for a long time no word appears in the color assigned to yourself. It is therefore important that you remain fully concentrated throughout the task, as words of your color may still appear at any time.

**In order to check that you are both concentrated over the entire duration of the task, each of you two will first indicate to which color category the word belongs by pressing a key for each word**. Please use the following index finger, middle finger, and ring finger of your right hand for the answers:

**Index finger = blue (= color category assigned to the person sitting on the left).**

**Middle finger = green (= color category not assigned to anyone)**

**Ring finger = red (= color category assigned to the person sitting on the right)**

If you are sitting on the right side of the computer, please use the "1", "2" and "3" keys on the Num Pad with your right hand for these answers: index finger ("1") for blue, middle finger ("2") for green, and ring finger ("3") for red.

If you are sitting on the left side of the computer, please use the keys "x", "c" and "v" with your right hand for these answers: index finger ("x") for blue, middle finger ("c") for green, and ring finger ("v") for red.

To summarize, there are three different events:

**1) A blue word appears:**

**- If you are sitting on the *left*, confirm by pressing the index finger that this word is blue**

**and memorize the word.**

**- If you are sitting on the *right*, press your index finger to confirm that this word is blue.**

**2) A green word appears:**

**- Regardless of whether you are sitting on the *left or right*: You confirm by pressing the button with your middle finger that this word is green.**

**3) A red word appears:**

**- If you are sitting on the *right,* press the ring finger to confirm that the word is red**

**and memorize the word.**

**- If you are sitting on the *left*, press the ring finger to confirm that this word is red.**

As mentioned, the keypresses to each word only serve to check your attention over time. It is more important that you always answer correctly, if possible, than that you answer quickly. It is also not important who of you answers faster. The most important point is to make as few mistakes as possible in your answers because this indicates optimal attention. If you still make a single mistake (wrong keypress or no keypress at all for a word), it is not consequential. Just continue to work in a concentrated manner and try to avoid further mistakes.

Note: The words will always remain on the screen for a certain additional amount of time after a keypress, i.e. a keypress does not cause the word to disappear. This is by design and does not mean that the keypresses were not registered.

The experimenter will now initially perform a practice run on the computer with both of you so that you are already familiar with the general experimental procedures when the actual run starts.

If you now have any questions about the procedure, please contact the experimenter.

**Supplementary Information S4:**

**Analyses with alternative categories (words from list positions 5 and 15 additionally included in the category “critical lures” to assess false memories)**

**Experiment 1**

In an alternative analysis, the 30 words from list positions 5 and 15 presented in the recognition memory task, were counted as “critical lures” (together with the original critical lures, i.e., the theme words from position 0 of the lists). Words from list positions 5 and 15 do not represent the semantic „culmination point“ of the respective list (common theme word to all words in a list), as the original critical lure does, but they still have some semantic association to the list words that were actually presented. Accordingly, in this alternative analysis, the category “critical lures” that assessed false memories encompassed 15 words per condition.

Regarding both overall false memories and only “remember” false memories, the overall discrimination parameter *P*_r_ was significantly higher than zero (*p*s ˂ .001), indicating genuine memories beyond a simple response bias (means and 95% CI for false overall memories: *FA*-CL = .36 [.34; .38], *FA*-D = .10 [.07; .13], *P*_r_ = .26 [.22; .30]; for false “remember” memories: *FA*-CL =.16 [.15; .18], *FA*-D = .03 [.02; .04], *P*_r_ = .13 [.10; .16]).

For the combined critical lures, the inclusion of the factor category led to better model fit, for both overall, likelihood ratio, $\chi^{2}$(2) = 160, *p* ˂ .001 and remember only responses, likelihood ratio, $\chi^{2}$(2) = 197, *p* ˂ .001. More false memories occurred for the self-assigned category, compared to the other two categories (all *p*s < .001). Importantly, there were more false memories in the other-assigned than in the non-assigned word category both in the overall memory analysis, *β* = 0.62, OR = 1.86 [CI 95%: 1.46; 2.39], *SE* = 0.13, *z* = 4.99, *p* < .001 and in only „remember“ answers, *β* = 0.56, OR = 1.76 [CI 95%: 1.21; 2.56], *SE* = 0.19, *z* = 2.94, *p* = .003. Means and standard deviations for the three experimental conditions are shown in Table S1.

For exploratory purposes, we also performed a separate analysis for the words from list positions 5 and 15 only (data also shown in Table S1). Including the factor category led to significant improvement, for both overall, likelihood ratio, $\chi^{2}$(2) = 51, *p* ˂ .001 and remember only responses, likelihood ratio, $\chi^{2}$(2) = 34, *p* ˂ .001. Again, the self-assigned category had the highest values for false memories (all *p*s < .001). For overall memory, there were more false memories in the other category, *β* = 0.61, OR = 1.85 [CI 95%: 1.34; 2.53], *SE* = 0.16, *z* = 3.82, *p* < .001, but not for only “remember” responses, *p* = .464.

**Table S1.** Data from alternative analyses of false memories in Experiment 1

|  | Overall false memories | | | |  | False memories - “remember” | | | | | |  | |
| --- | --- | --- | --- | --- | --- | --- | --- | --- | --- | --- | --- | --- | --- |
|  | Critical Lures  incl. Pos. 5&15 | | Only words from Pos. 5&15 | |  | Critical Lures  incl. Pos. 5&15 | | Only words from Pos. 5&15 | | |  |  |  |
|  | *M* | *SD* | *M* | *SD* | *M* | | *SD* | | *M* | *SD* | | |  |
| Self | .51 | .50 | .36 | .48 | .29 | | .45 | | .15 | .36 | | |  |
| Other | **.34**** | .47 | .27** | .44 | **.11**** | | .32 | | .07 | .25 | | |  |
| None | **.24** | .43 | .18 | .39 | **.08** | | .27 | | .06 | .24 | | |  |

Note: Data indicate mean proportions (*M*) and standard deviations (*SD*) for "Yes" answers in the recognition memory test (left panel) and for only those "Yes" answers with additional indication of a “remember” experience (right panel), separately for the three word categories (Self, Other, None). All “Self” means differ significantly from the corresponding means in the other two categories (all *p*s < .001). Critical statistical comparisons (shown in bold) refer to differences in the means of all critical lures between the two categories “Other” and “None”. **^**^** *p* < .01, for difference between “Other” and “None”.

**Experiment 2**

As in Experiment 1, the 30 words from list positions 5 and 15 presented in the recognition memory task, were in an alternative analysis counted as “critical lures” (together with the original critical lures, i.e., the theme words from position 0 of the lists). Regarding both overall false memories and false memories with additional corresponding source memory, the overall discrimination parameter *P*_r_ was significantly higher than zero (*p*s ˂ .001), indicating genuine memories beyond a simple response bias (means and 95% CI for false overall memories: *FA*-CL = .46 [.44; .48], *FA*-D = .22 [.19; .25], *P*_r_ = .24 [.20; .28]; for false memories with additional source memory: *FA*-CL =.33 [.32; .35], *FA*-D = .01 [.00; .01], *P*_r_ = .32 [.29; .35]).

For the combined lures, the inclusion of the factor category led to improved model fit, for both overall, likelihood ratio, $\chi^{2}$(2) = 100, *p* ˂ .001 and memories with additionally remembered source, likelihood ratio, $\chi^{2}$(2) = 141, *p* ˂ .001. More false memories were found for the self-assigned category (all *p*s < .001). Critically, there were more false memories in the other-assigned than in the non-assigned word category both in the overall memory analysis, *β* = 0.49, OR = 1.63 [CI 95%: 1.31; 2.02], *SE* = 0.11, *z* = 4.44, *p* < .001 and memories with additional source memory, *β* = 0.51, OR = 1.67 [CI 95%: 1.32; 2.10], *SE* = 0.12 , *z* = 4.31 , *p* < .001. Means and standard deviations for the three experimental conditions are shown in Table S2.

For exploratory purposes, we again also performed a separate analysis for the words from list positions 5 and 15 only (data also shown in Table S2). Including the factor category led to significant improvement, for both overall, likelihood ratio, $\chi^{2}$(2) = 32, *p* ˂ .001 and memories with additional source memory, likelihood ratio, $\chi^{2}$(2) = 29, *p* ˂ .001. The self-assigned category again had the highest values compared to the other two categories (*p*s < .001). More false memories were found in the other-assigned than in the non-assigned category, both overall, *β* = 0.51, OR = 1.67 [CI 95%: 1.27; 2.18], *SE* = 0.14, *z* = 3.72, *p* < .001, and for false memories with additionally remembered source, *β* = 0.46, OR = 1.58 [CI 95%: 1.18; 2.13], *SE* = 0.15, *z* = 3.01, *p* < .001.

**Table S2.** Data from alternative analyses of false memories in Experiment 2

|  | Overall false memories | | | |  | False memories - “remember” | | | | | |  | |
| --- | --- | --- | --- | --- | --- | --- | --- | --- | --- | --- | --- | --- | --- |
|  | Critical Lures  incl. Pos. 5&15 | | Only words from Pos. 5&15 | |  | Critical Lures  incl. Pos. 5&15 | | Only words from Pos. 5&15 | | |  |  |  |
|  | *M* | *SD* | *M* | *SD* | *M* | | *SD* | | *M* | *SD* | | |  |
| Self | .57 | .49 | .44 | .50 | .46 | | .50 | | .31 | .46 | | |  |
| Other | **.45**** | .50 | .39** | .49 | **.31**** | | .46 | | .25** | .43 | | |  |
| None | **.36** | .48 | .30 | .46 | **.23** | | .42 | | .18 | .39 | | |  |

Note: Data indicate mean proportions (*M*) and standard deviations (*SD*) for "Yes" answers in the recognition memory test (left panel) and for only those "Yes" answers with additional indication of respective source memory (right panel), separately for the three word categories (Self, Other, None). All “Self” means differ significantly from the corresponding means in the other two categories (all *p*s < .001). Critical statistical comparisons (shown in bold) refer to differences in the means of all critical lures between the two categories “Other” and “None.

**^**^** *p* < .01, for difference between “Other” and “None”.
